# *In Silico* Mining of Natural Products Atlas (NPAtlas) Database for Identifying Effective Bcl-2 Inhibitors: Molecular Docking, Molecular Dynamics, and Pharmacokinetics Characteristics

**DOI:** 10.3390/molecules28020783

**Published:** 2023-01-12

**Authors:** Nahlah Makki Almansour, Khaled S. Allemailem, Abeer Abas Abd El Aty, Ekram Ismail Fagiree Ismail, Mahmoud A. A. Ibrahim

**Affiliations:** 1 Department of Biology, College of Science, University of Hafr Al Batin, Hafr Al Batin 1803, Saudi Arabia; 2 Department of Medical Laboratories, College of Applied Medical Sciences, Qassim University, Buraydah 51452, Saudi Arabia; 3 Computational Chemistry Laboratory, Chemistry Department, Faculty of Science, Minia University, Minia 61519, Egypt; 4 School of Health Sciences, University of KwaZulu-Natal, Westville, Durban 4000, South Africa

**Keywords:** Bcl-2 protein, cancer disease, MD simulations, molecular docking, pharmacokinetics features

## Abstract

The Bcl-2 protein has a vital function in controlling the programmed cell doom of mitochondria. If programmed cell death signals are obstructed, an imbalance between cell survival and death will occur, which is a significant reason for cancer. Therefore, the Bcl-2 protein was identified as a possible therapeutic target for carcinoma treatment. Herein, the Natural Products Atlas (NPAtlas) compounds were virtually screened, seeking potent inhibitors towards the Bcl-2 protein. The performance of AutoDock Vina software to predict the docking score and pose of the investigated compounds was first validated according to the available experimental data. Based on the validated AutoDock Vina parameters, the NPAtlas database was filtered against the Bcl-2 protein. The natural compounds with docking scores less than that of the venetoclax (calc. −10.6 kcal/mol) were submitted to MD simulations, followed by MM-GBSA binding energy calculations. According to MM-GBSA//200 ns MD simulations, saquayamycin F (NPA002200) demonstrated promising binding affinity with a Δ*G*_binding_ value of −53.9 kcal/mol towards the Bcl-2 protein when compared to venetoclax (Δ*G*_binding_ = −50.6 kcal/mol). The energetical and structural analyses showed a great constancy of the saquayamycin F inside the Bcl-2 protein active site. Moreover, the ADMET and drug-likeness features of the saquayamycin F were anticipated, indicating its good oral bioavailability. According to *in silico* computations, saquayamycin F is proposed to be used as a therapeutic agent against the wild-type Bcl-2 protein and warrants further experimental assays.

## 1. Introduction

Carcinoma is a severe malady and the second most common reason for mortality [1]. Globally, millions of people receive cancer diagnoses annually, and more than half of those individuals ultimately pass away from the disease [2]. Dysregulation of the normal apoptotic processes in living cells may cause cancer disease [3]. Apoptosis can be defined as a regulated process required for the elimination of damaged or aged cells [3]. The counterbalance between pro-apoptotic and anti-apoptotic of the Bcl-2 (B-cell lymphoma-2) family has a substantial role in the apoptosis process [4,5,6,7]. The anti-apoptotic Bcl-2 family involves BFL-1/A1, BCL-XL, BCL-2, and MCL-1 proteins [8]. Comprehensive directories indicate that overexpression of anti-apoptotic Bcl-2 protein may be noticed in many cancer cells, and its overexpression is the fundamental cause of neoplasm cells to avoid apoptosis and gain resistance to chemotherapy [9]. Therefore, the regulation of the anti-apoptotic Bcl-2 protein has a critical function in treating carcinoma. A small number of inhibitors against Bcl-2 protein have been developed, such as obatoclax [10], venetoclax [11], and navitoclax [12]. Venetoclax was approved in April 2016 by the Food and Drug Administration (FDA) to cure CLL (chronic lymphocytic leukemia). Venetoclax was designed by reverse engineering the navitoclax to increase the Bcl-2 selectivity demonstrating a ten-fold higher potency against CLL cells [13].

Natural products (NPs) obtained from plants and microbes are crucial in developing new medicines, especially for treating cancer and infectious diseases [14,15]. A large number of medications are NPs from plants, such as morphine, paclitaxel, and vinblastine [16]. Hence, there is a considerable chance for exploring novel anticarcinoma drug candidates as wild-type Bcl-2 inhibitors from the Natural Products Atlas (NPAtlas) database. In this work, the NPAtlas database, containing >24,500 natural compounds, was virtually screened for any promising Bcl-2 inhibitors using AutoDock Vina software [17]. After that, the most potent compounds were nominated and submitted for molecular dynamics simulations. The corresponding binding energies of the NPAtlas-Bcl-2 complexes were computed using the MM-GBSA approach. The post-MD analyses of the most promising compounds complexed with the Bcl-2 protein were conducted. The physicochemical and pharmacokinetic features of the most promising compounds were also demonstrated. Consequently, these findings shine a new light on the prospective compounds as wild-type Bcl-2 inhibitors and present a viable therapeutic candidate for experimental investigations.

## 2. Results and Discussion

### 2.1. Docking Assessment

**Figure 1:**
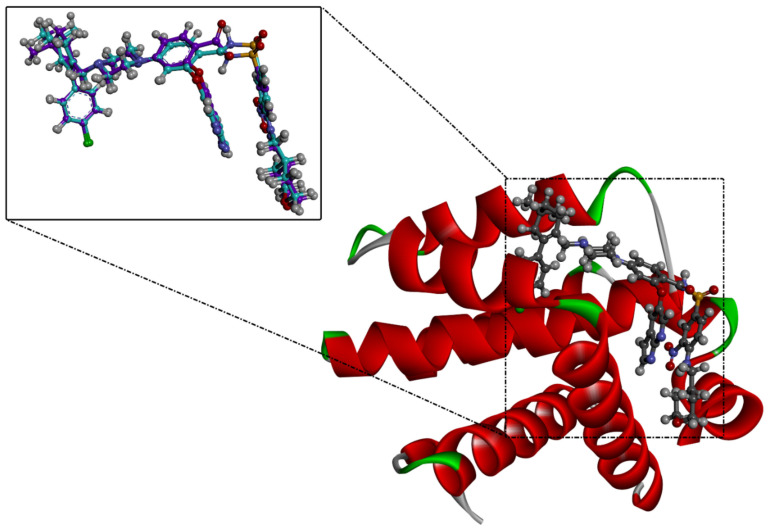
Three-dimensional superimposition of the resolved experimental structure (in mauve) and the anticipated binding mode (in cyan) of venetoclax complexed with Bcl-2 protein.

Prior to identifying potent wild-type Bcl-2 inhibitors using a molecular docking technique, the performance of the employed docking protocol was first assessed. The co-crystallized venetoclax ligand was re-docked towards Bcl-2 protein using Autodock Vina software, and the predicted docking pose was compared to the native pose (PDB access code: 6O0K [18]) (Figure 1). As illustrated in Figure 1, the anticipated docking pose was approximately identical to the native binding mode with an RMSD of 0.21 Å. Furthermore, these findings emphasize the accuracy of this technique in hunting potent compounds as potential Bcl-2 inhibitors.

### 2.2. NPAtlas Database Screening

The NPAtlas database was virtually screened towards the Bcl-2 protein utilizing fast docking computations (see Methodology section for details). Based on the predicted docking scores, only 56 compounds displayed docking scores lower than that of the venetoclax inhibitor (calc. −10.6 kcal/mol) towards the Bcl-2 protein. Therefore, those 56 compounds underwent a more reliable docking computation with expensive docking parameters (see Methodology section for details). The expensive docking scores for the most promising 56 compounds were estimated and listed in Table S1. From Table S1, 42 compounds displayed docking scores lower than that of venetoclax (calc. −10.6 kcal/mol). The computed docking scores and the 2D chemical structures for these 42 compounds with the Bcl-2 protein are summarized in Table 1. The 2D molecular interactions of the binding modes for these compounds are presented in Figure S1. Concerning Figure S1, the 2D molecular interactions manifested hydrogen bonding of these compounds with GLY145, ASN143, TRP144, and ARG146 residues within the Bcl-2 active site. Hydrophobic, π-based, and vdW interactions were ditto observed between the most important residues within the Bcl-2 binding pocket and the investigated compounds (Figure S1).

Saquayamycin F (NPA002200) is a prospective inhibitor of the farnesyl protein transferase (FPTase) with an IC_50_ value of 2.0 μM [19], revealing the best docking score against the Bcl-2 protein with a value of −12.0 kcal/mol (Table 1). It has also been reported that saquayamycins demonstrate anticancer and antibacterial activities in a sub-micromolar range [20,21]; additionally, a saquayamycin analog (saq. B1) showed inhibitory activity towards the PI3K/AKT signaling pathway, including Bcl-2, in human colorectal cancer cells [22].

**Figure 2:**
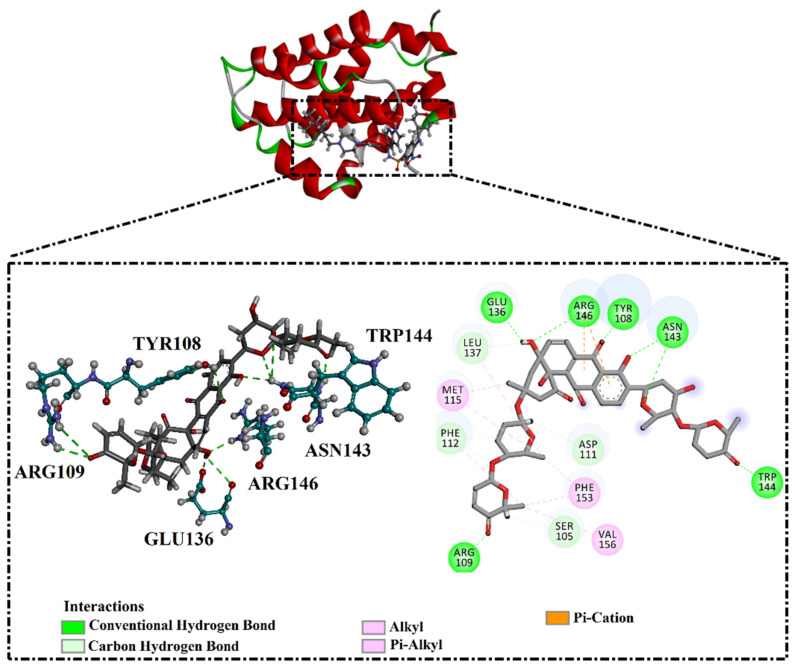
The three- and two-dimensional molecular interactions of saquayamycin F (NPA002200) complexed with the Bcl-2 protein.

Inspecting the binding mode of saquayamycin F inside the binding pocket of the Bcl-2 protein demonstrated seven H-bonds with key residues of the Bcl-2 protein (Figure 2). The oxygen atom of 2-methyl-2H-pyran-3(6H)-one group exhibited a H-bond with the NH2 group of ARG109 residue with a bond length of 2.44 Å (Figure 2). The OH group of the dihydroxy-5-methylcycohexan-1-one group established two H-bonds with the NH2 group and oxygen atom of the ARG146 and GLU136 residues with bond lengths of 2.14 and 1.92 Å, respectively (Figure 2). The oxygen atom of cyclohex-2-ene-1,4-dione group demonstrated a hydrogen bond with the OH group of the TYR108 residue with a bond length of 2.74 Å (Figure 2). The ASN143 residue formed two H-bonds with the oxygen atom of the phenol ring and the tetrahydro-2H-pyran-4-ol group with bond lengths of 2.45 and 3.2 Å, respectively (Figure 2). Finally, the oxygen atom of dihydro-2H-pyran-3(4H)-one group displayed a H-bond with the OH group of the TRP144 residue with a bond length of 1.90 Å (Figure 2).

### 2.3. Molecular Dynamics Simulations

**Figure 3:**
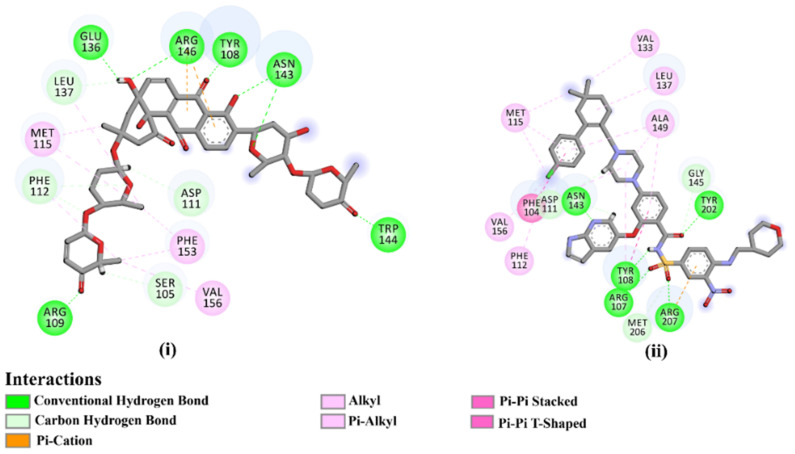
Two-dimensional molecular interactions of (**i**) saquayamycin F (NPA002200) and (**ii**) venetoclax with the Bcl-2 protein according to the final snapshot over 50 ns MD simulations.

As the receptor-inhibitor complexes retrieved from docking computations are stationary entities, consequently conducting molecular dynamics (MD) simulations is considered to be a pivotal portion of any *in silico* study [23]. The MD simulations provide elaborate results with regard to the receptor-inhibitor interactions with a dynamic aspect [24,25]. The MD simulations are executed to realize more in depth insights into the influence of receptor flexibility and structural changes of the investigated complexes [24,26]. Therefore, the most potent NPAtlas (42 compounds with docking scores <−10.6 kcal/mol) complexed with the Bcl-2 protein were subjected to the MD simulations over 50 ns. The corresponding binding affinities of those compounds with the Bcl-2 protein were also estimated and are compiled in Table S2. As listed in Table S2, only saquayamycin F (NPA002200) manifested binding affinity, which was less than that of venetoclax towards the Bcl-2 protein with Δ*G*_binding_ values of −48.1 and −46.0 kcal/mol, respectively. The 2D and 3D representations of the final snapshots of saquayamycin F and venetoclax within the Bcl-2 active site throughout 50 ns MD simulations are presented in Figure 3 and Figure S2, respectively. Investigating the binding mode of saquayamycin F demonstrated that saquayamycin F conserved its hydrogen bonds with ARG109, TYR108, GLU136, ASN143, TRP144, and ARG146 residues (Figure 3 and Figure S2). For venetoclax complexed with the Bcl-2 protein, the NH and CO of the amide group exhibited two H-bonds with the OH groups of TYR108 and TYR202 residues with bond lengths of 1.86 and 1.77 Å, respectively (Figure 3 and Figure S2). Moreover, the sulfonyl group formed two H-bonds with NH2 and NH groups of ARG107 and ARG207 residues with bond lengths of 2.01 and 2.17 Å, respectively (Figure 3 and Figure S2). In addition, the nitrogen atom of the pyridine ring interacted with the NH2 of ASN143 residue by an H-bond with a bond length of 2.09 Å (Figure 3 and Figure S2).

Regarding the more reliable results, the MD simulations for saquayamycin F and venetoclax in complex with the Bcl-2 protein were elongated to 200 ns. The MM-GBSA binding energies were then computed and are summarized in Table 2. Based on the data presented in Table 2, no tangible disparity between the evaluated binding affinities for the saquayamycin F complexed with the Bcl-2 protein over 50 and 200 ns MD simulations was noticed. When compared to the venetoclax inhibitor (calc. −51.2 kcal/mol), saquayamycin F disclosed a higher binding affinity towards the Bcl-2 protein during the 200 ns MD simulations with an average Δ*G*_binding_ value of −53.9 kcal/mol (Table 2).

**Figure 4:**
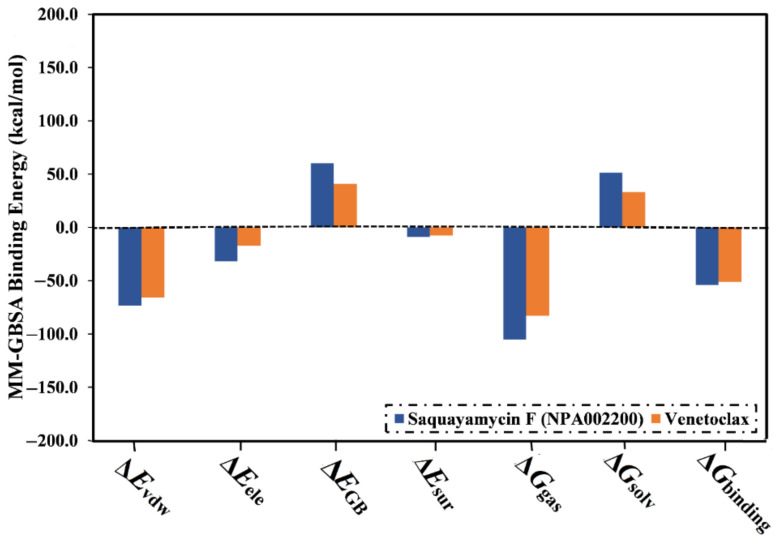
Binding energy components for venetoclax and the saquayamycin F (NPA002200) complexed with the Bcl-2 protein during the 200 ns MD simulations.

The computed binding affinities were decomposed into individual elements to scout the driving forces in the binding of saquayamycin F (NPA002200) and venetoclax with the Bcl-2 protein (Figure 4). From Figure 4, the binding energy of saquayamycin F and venetoclax with the Bcl-2 protein were dominated by the van der Waals forces (*E*_vdw_) with average values of −73.4 and −65.7 kcal/mol, respectively (Figure 4). Moreover, the electrostatic forces (*E*_ele_) were a favorable contributor to the binding energies of saquayamycin F and venetoclax with the Bcl-2 protein, with values of −31.8 and −17.3 kcal/mol. It is worth noting that *E*_vdw_ is about two and a half folds more vigorous when compared to *E*_ele_.

**Figure 5:**
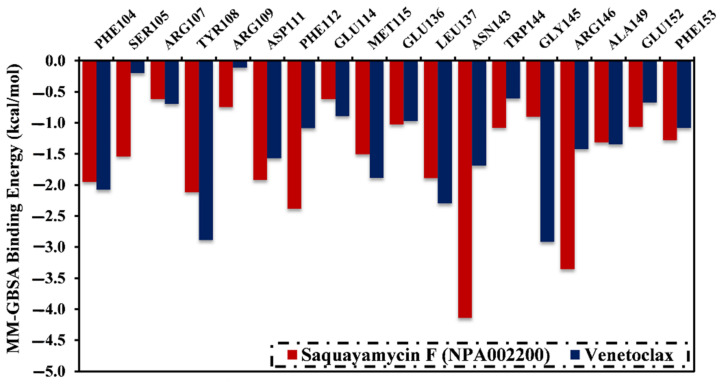
The contribution of the most favorable amino acids to the total binding energy (kcal/mol) of saquayamycin F (NPA002200) and venetoclax with the Bcl-2 protein over 200 ns MD simulations.

A per-residue energy decomposition analysis was also conducted to elucidate the key residues that participate in the interactions of saquayamycin F- and venetoclax-Bcl-2 complexes (Figure 5). The residues with energy contributions lower than −0.5 kcal/mol are given in Figure 5. As depicted in Figure 5, per-residue energy decomposition analysis displayed that the ASN143, TYR108, and ARG146 residues interacted with saquayamycin F and venetoclax. A considerable participation of the ASN143 residue to the total binding energies was noticed with values of −4.1 and −1.9 kcal/mol for saquayamycin F- and venetoclax-Bcl-2 complexes, respectively (Figure 5).

### 2.4. Post-Dynamics Analyses

The steadiness and nature of the interactions of saquayamycin F (NPA002200) inside the Bcl-2 binding pocket were explained using the post-MD analyses. The energetical and structural analyses for saquayamycin F and venetoclax were executed throughout the 200 ns MD simulations. The post-MD analyses involved a binding affinity analysis, H-bond analysis, the CoM, RMSD, Rg, and RMSF.

#### 2.4.1. Binding Affinity Analysis

**Figure 6:**
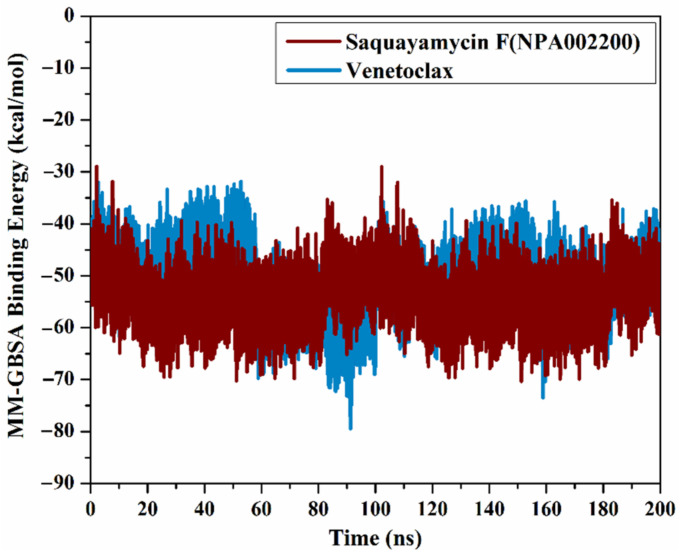
The evaluated MM-GBSA binding energies of saquayamycin F (NPA002200) (in dark red) and venetoclax (light blue) with the Bcl-2 protein throughout the 200 ns MD course.

The correlation between the estimated binding affinity and the time was executed to detect the energetical constancy of saquayamycin F (NPA002200) and venetoclax inside the Bcl-2 active site over 200 ns MD simulations (Figure 6). From Figure 6, saquayamycin F and venetoclax were generally stable until the termination of the simulations with Δ*G*_binding_ values of −53.8 and −50.6 kcal/mol, respectively. These results revealed the great steadiness of saquayamycin F and venetoclax with the Bcl-2 protein throughout the 200 ns MD course.

#### 2.4.2. H-Bond Analysis

**Figure 7:**
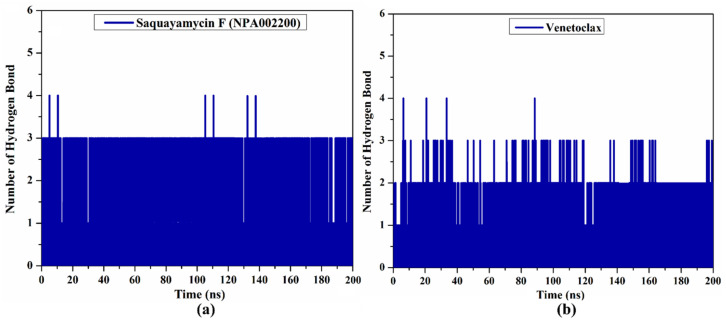
The number of hydrogen bonds over 200 ns MD simulations for (**a**) saquayamycin F (NPA002200) and (**b**) venetoclax complexed with the Bcl-2 protein.

Because the H-bond number is linked to binding strength, the intermolecular H-bond number between saquayamycin F (NPA002200) and venetoclax and the Bcl-2 protein during the 200 ns MD course is depicted in Figure 7. As illustrated in Figure 7, the average H-bond number was three and two for saquayamycin F and venetoclax complexed with the Bcl-2 protein over the 200 ns MD simulations. From these observations, saquayamycin F seems more tightly bound to the Bcl-2 protein than the co-crystallized venetoclax inhibitor.

#### 2.4.3. CoM Distance

**Figure 8:**
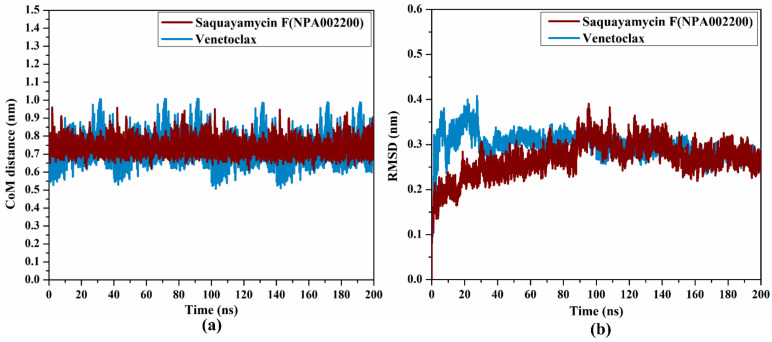
(**a**) CoM distance and (**b**) RMSD of the backbone atoms from the starting structure of saquayamycin F (in dark red) and venetoclax (light blue) in complex with the Bcl-2 protein during the 200 ns MD course.

To obtain a deeper insight into the stability of saquayamycin F (NPA002200) and venetoclax in complex with the Bcl-2 protein during the simulation of 200 ns, the center-of-mass (CoM) distance was gauged between the inspected inhibitors and the ASN143 residue (Figure 8a). As shown in Figure 8a, the greater stability of saquayamycin F-Bcl-2 complex was observed when compared to venetoclax-Bcl-2 complex with average CoM distances of 0.8 and 0.9 nm, respectively (Figure 8a). These findings reflect the constancy of saquayamycin F, which further confirms that saquayamycin F tends to bind strongly with the Bcl-2 protein, as previous analyses manifested.

#### 2.4.4. RMSD Analysis

To examine if there are any conformational variations of the Bcl-2 protein after complexation with saquayamycin F and venetoclax, the root-mean-square deviation (RMSD) analysis was inspected over the 200 ns MD simulations (Figure 8b). From Figure 8b, the average RMSD values were 0.2 and 0.31 nm for saquayamycin F and venetoclax complexed with the Bcl-2 protein, respectively. The RMSD analysis demonstrated that the inspected complexes attained a stable conformation after 20 ns until the termination of the simulation. These findings confirmed that saquayamycin F is tightly bonded and does not impact the structural steadiness of the Bcl-2 protein.

#### 2.4.5. RMSF Analysis

**Figure 9:**
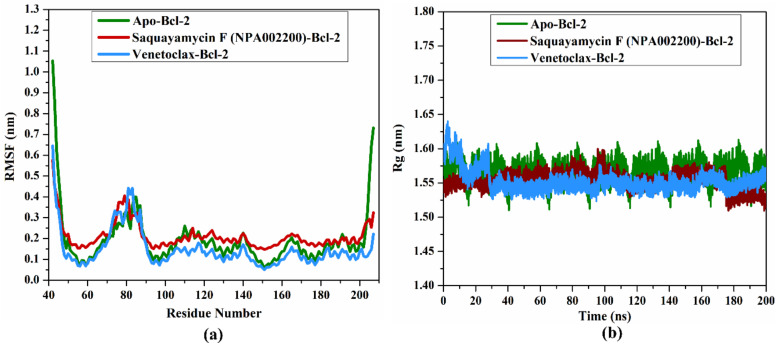
(**a**) RMSF of the C_α_ atoms and (**b**) Rg of apo- and inhibitor-soaked Bcl-2 protein during 200 ns MD course. Color Scheme: Apo-Bcl-2 protein (in green), saquayamycin F-Bcl-2 (in dark red), and venetoclax-Bcl-2 (light blue).

In order to estimate the variance of the apo-Bcl-2 protein, saquayamycin F-Bcl-2, and venetoclax-Bcl-2 complexes throughout the 200 ns MD simulations, the root-mean-square fluctuation (RMSF) analysis of alpha carbon was computed and depicted in Figure 9a. Upon the data demonstrated in Figure 9a, the average RMSF values were 0.19, 0.21, and 0.14 nm for the apo-Bcl-2 protein, saquayamycin F-Bcl-2, and venetoclax-Bcl-2 complexes, respectively. The RMSF plot demonstrated that the apo-Bcl-2 protein, saquayamycin F-Bcl-2, and venetoclax-Bcl-2 complexes remained stable over the 200 ns MD simulations (Figure 9a).

#### 2.4.6. Radius of Gyration

The radius of gyration (Rg) was estimated to observe the compactness of the Bcl-2 protein in the apo- and ligand-soaked forms during the 200 ns MD simulations. According to Figure 9b, the average Rg values were 1.57, 1.53, and 1.55 nm for the apo-Bcl-2 protein, saquayamycin F-Bcl-2, and venetoclax-Bcl-2 complexes, respectively. Overall, the Rg analysis confirmed that the complexation of the Bcl-2 protein with saquayamycin F and venetoclax significantly stabilized the Bcl-2 structure.

### 2.5. Physicochemical Properties

The knowledge of physicochemical properties provides useful guidelines for primary-phase drug discovery. Usually, the most known Lipinski’s rule of five is employed as an evaluation for physicochemical properties of prospective durg candidates. For saquayamycin F (NPA002200), miLog*P* value was lower than 5 (calc. 0.65), demonstrating its great lipophilicity. However, the miLog*P* value of venetoclax was greater than 5 (calc. 8.43). The MW of saquayamycin F and venetoclax were greater than 500 dalton (Table 3). For saquayamycin F and venetoclax, nON was greater than 10. Notably, these violations in MW and nON do not substantially influence the molecule transportation, where a large number of FDA-approved drugs sidetracked from the nON of 10 and MW of 500 [27]. Otherwise, the number of nOHNH was less than 5 for saquayamycin F and venetoclax (Table 3). The TPSA values of saquayamycin F and venetoclax were 230.91 and 174.72 Å^2^, respectively (Table 3). Ultimately, the evaluated %ABS of saquayamycin F and venetoclax were 29.3% and 48.7%, respectively.

### 2.6. Pharmacokinetic Features

Anticipating human pharmacokinetic features is vital for the drug discovery process since it assists in identifying the drug candidates that can be the most successful in clinical investigations [28]. Therefore, the ADMET properties of saquayamycin F (NPA002200) and venetoclax were evaluated. The Caco2 permeability and HIA are substantial absorption properties in drug discovery [29]. Both saquayamycin F and venetoclax demonstrated low Caco2 permeability with values of 0.271 and 0.847, respectively (Table 4). Moreover, saquayamycin F demonstrated a lower percentage of HIA (calc. 80.9%) compared to venetoclax (calc. 100%) (Table 4). The BBB membrane permeability was utilized to examine the distribution property. For BBB, log BB values should be in the range of <−1 to >0.3. Both saquayamycin F and venetoclax were anticipated to be not able to cross the BBB membrane (Table 4). The CYP450 plays an essential function in drug metabolism in the liver [30]. The metabolism anticipation displayed that saquayamycin F and venetoclax were CYP3A4 inhibitors/substrates. Total clearance determines the drug concentration in the human body [31]. The total clearance ranged from 0 to 1.0 mL/min/kg. The measured outcomes manifested that the total clearance for saquayamycin F and venetoclax was −0.196 and −0.096 mL/min/kg, respectively (Table 4). Toxicity has a significant function in the choosing of the most appropriate drugs in the drug development process. Both saquayamycin F and venetoclax did not expressed any AMES toxicity (Table 4).

## 3. Computational Methodology

### 3.1. Bcl-2 Preparation

The wild-type Bcl-2 crystal structure complexed with the venetoclax ligand was downloaded (PDB code: 6O0K; resolution: 1.62 Å [18]) and selected as a template for all *in silico* estimations. The missing residues of the Bcl-2 protein were built using Modeller software [32]. The ions, inhibitor, and water molecules were removed from the Bcl-2 protein. The protonation state of the Bcl-2 protein was then assigned by the H++ server [33]. All the missing hydrogens were also inserted.

### 3.2. Database Preparation

**Figure 10:**
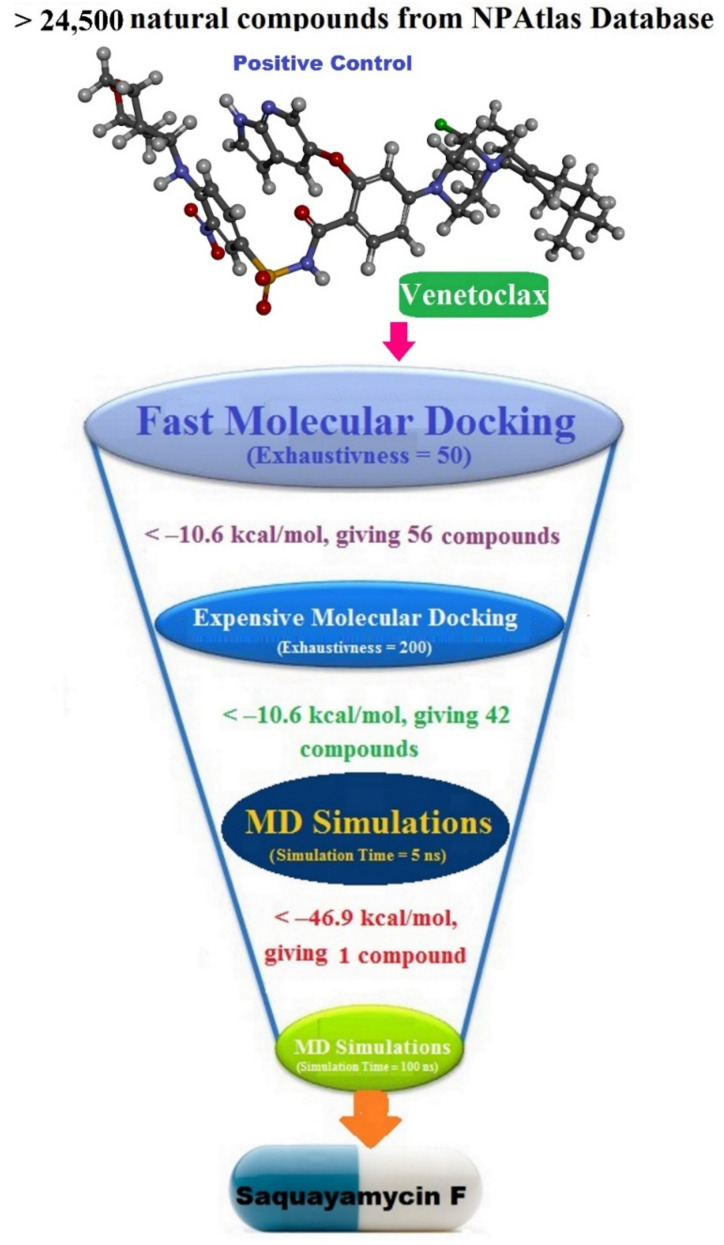
Schematic representation of the utilized *in silico* techniques in the filtration of the NPAtlas database towards identification of promising Bcl-2 inhibitors.

The NPAtlas (Natural Products Atlas) database, containing >24,500 natural molecules, was downloaded in SDF format [17]. The duplicated compounds were eliminated based on the International Chemical Identifier (InChIKey) [34]. Omega2 software was applied to generate the 3D chemical structures [35,36]. Thereafter, the MMFF94S force field within SZYBKI software was employed to minimize the generated compounds [37,38]. All the prepared files can be found in the CompChem database (www.compchem.net/ccdb, accessed on 1 December 2022). Figure 10 illustrates the workflow of the utilized computational techniques in the virtual screening process of the database.

### 3.3. Docking Computations

AutoDock Vina1.1.2 software was applied to execute all the docking computations [39]. The Bcl-2 protein was converted into pdbqt format utilizing MGL1.5.7 tools [40], which is required for this software. The exhaustiveness number was adjusted to 50 and 200 for fast and expensive docking predictions, respectively. All remaining parameters were maintained at their default values. The docking grid size was 25 Å × 25 Å × 25 Å, with a spacing value of 1 Å. The grid was located at −17.329, 3.971, −11.668 (in x, y, and z dimensions, respectively).

### 3.4. MD Simulations

All molecular dynamic (MD) simulations were conducted using AMBER16 software [41]. The specifics of the MD simulations are characterized elsewhere [42,43,44,45]. Briefly, the AMBER force field of 14SB was used to characterize the Bcl-2 protein [46]. The GAFF2 (general AMBER force field) was employed to parameterize the most potent compounds [47]. Utilizing Gaussian09 software [48], the investigated compounds were optimized at HF/6-31G* level of theory for atomic charge calculations. The atomic charges of the optimized compounds were assigned using the RESP (restricted electrostatic potential) approach [49]. TIP3P water molecules in an octahedron box with a value of 12 Å at the box edge from any atom in the system were employed to solvate the NPAtlas-Bcl-2 complexes. To neutralize the NPAtlas-Bcl-2 complexes, the Cl^−^/Na^+^ counterions were inserted. Additionally, the NaCl concentration was set to 0.15 M. The solvated complexes were energetically minimized for 5000 cycles. The minimized complexes were gradually warmed to 300 K over 50 ps. Furthermore, the complexes were equilibrated over 10 ns under the NPT ensemble. The equilibrated coordinates of these complexes were subjected to production phases over 50 and 200 ns MD simulations. The trajectories were gathered every 10 ps. Using the PME (Particle Mesh Ewald) method, the electrostatic interactions were handled [50]. The Berendsen barostat was applied to preserve the pressure [51]. The barostat relaxation time was adjusted to 2 ps. All the atomic hydrogen bonds were constrained by the SHAKE algorithm [52], and the integration time step of 2 fs was employed. The pmemd.cuda supplied by the AMBER16 package was utilized to run the MD simulations on the CompChem GPU/CPU hybrid cluster (hpc.compchem.net). All the molecular interactions were represented using the BIOVIA Materials Studio [53].

### 3.5. Binding Energy Computations

The binding affinities of the most promising NPAtlas-Bcl-2 complexes were computed using the MM-GBSA (molecular mechanics-generalized Born surface area) approach [54]. Polar solvation energy was specified utilizing the modulated GB model suggested by Onufriev (igb  =  2) [55]. The binding affinity was estimated by the following mathematic equation:

$$\Delta G_{\mathrm{binding}}=G_{\mathrm{Complex}\;}-\left(G_{\mathrm{NPAtlas}}+G_{\mathrm{Bcl}-2}\right)$$

where the *G* term is:

$$G=G_{\mathrm{GB}}+G_{\mathrm{SA}}+E_{\mathrm{ele}}+E_{\mathrm{vdw}}$$

*G*_GB_ implies the electrostatic solvation free energy. *G*_SA_ indicates the nonpolar solvation-free energy. *E*_ele_ is electrostatic energy. *E*_vdw_ refers to van der Waals energy. Because of the high computational costs and time, the entropy (*S*) estimations were not estimated [56,57].

### 3.6. Physicochemical Properties

The physicochemical features of the identified compound were pointed out by Lipinski’s rule of five (RO5) parameters using the online Molinspiration cheminformatics software (http://www.molinspiration.com, accessed on 1 December 2022). Based on the RO5, the orally active drug must have no more than one violation of the following criteria: no more than 10 hydrogen bond acceptors (nON), a number of hydrogen bond donors (nOHNH) should be <5, the molecular weight (MW) should be <500 Da, the partition coefficient logP (miLog*P*), and the topological polar surface area (TPSA) should not be greater than 5 and 140 Å2 [58].

### 3.7. Pharmacokinetic Features

The ADMET (Absorption, distribution, metabolism, excretion, and toxicity) characteristics were predicted for the identified NPAtlas compounds using the pkCSM web server (http://biosig.unimelb.edu.au/pkcsm/prediction, accessed on 1 December 2022). The absorption (A) was predicted based on the Caco2 permeability and the HIA (human intestinal absorption) characteristics. The BBB (Blood-brain barrier) permeability and the CYP2D6/CYP3A4 substrate were employed to predict the distribution (D) and the metabolism (M). Drug clearance was used to foretell the excretion (E) feature. The toxicity (T) was predicted via the AMES toxicity.

## 4. Conclusions

The Bcl-2 protein is a promising therapeutic target for treating carcinoma disease because of its capacity to control the programmed cell death of mitochondria. Hence, this work was accomplished by a combination of docking predictions and MD simulations to identify potent Bcl-2 inhibitors from a natural source. Therefore, the NPAtlas database, containing more than 24,500 natural compounds, was filtered toward the Bcl-2 binding pocket using AutoDock Vina software. Furthermore, the most potent compounds were submitted to 50 and 200 ns MD simulations, and their corresponding binding energies were computed using the MM-GBSA approach. According to the current outcomes, saquayamycin F (NPA002200) disclosed an outstanding binding energy with an average Δ*G*_binding_ of −53.8 kcal/mol when compared to venetoclax (calc. −50.6 kcal/mol). The steadiness of the saquayamycin F inside the Bcl-2 binding pocket was confirmed by the post-MD analyses of over the 200 ns MD simulations. Saquayamycin F also demonstrated appropriate physicochemical and pharmacokinetic characteristics. Eventually, the current results demonstrated that saquayamycin F may be an anticarcinoma drug candidate and warrants additional experimental investigations.

## Figures and Tables

**Table 1:** Two-dimensional chemical structures and expected fast and expensive vina docking scores (in kcal/mol) for the top 42 potential NPAtlas compounds against the Bcl-2 protein compared to venetoclax ^a^.

Compound Name/Code	Two-Dimensional Chemical Structure	Docking Score (kcal/mol)		Compound Name/Code	Two-Dimensional Chemical Structure	Docking Score (kcal/mol)	
		Fast	Expensive			Fast	Expensive
Venetoclax	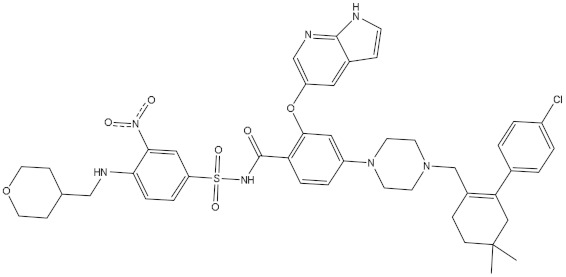	−10.6	−10.6	NPA018272 (Landomycin Y)	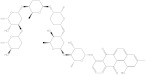	−11.3	−11.4
NPA002200 (Saquayamycin F)	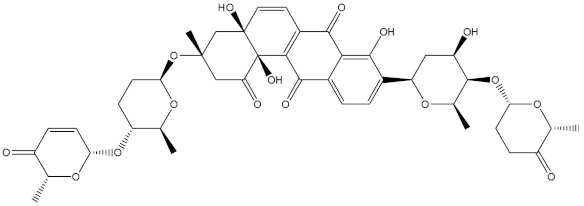	−12.0	−12.0	NPA012375 (VM-55595)	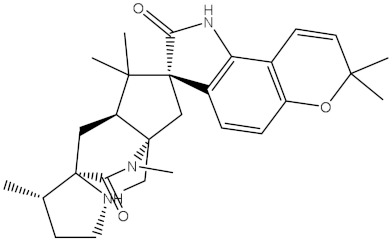	−11.4	−11.4
NPA032668 (Landomycin M)	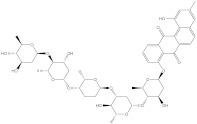	−11.7	−11.8	NPA008326 (N-hydroxy-6-epi-stephacidin A)	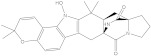	−11.3	−11.3
NPA004880 (Saquayamycin E)	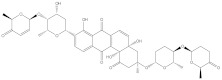	−11.7	−11.7	NPA025253 (Shearilicine)	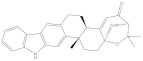	−11.3	−11.3
NPA021302 (Paraherquamide F)	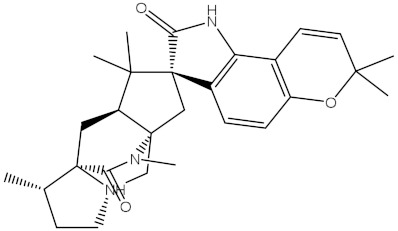	−11.6	−11.0	NPA019494 (Landomycin T)	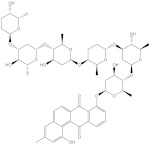	−11.2	−11.2
NPA008122 (Naseseazine C)	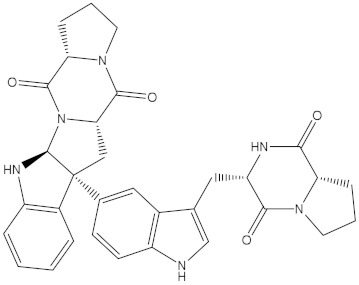	−11.5	−11.5	NPA005301 (Marcfortine C)	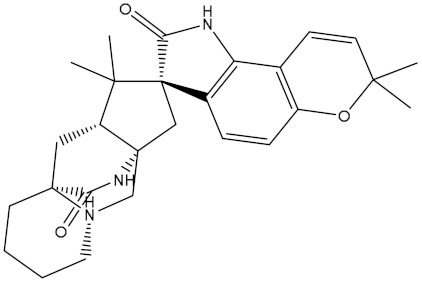	−11.1	−11.2
NPA005183 (Landomycin W)	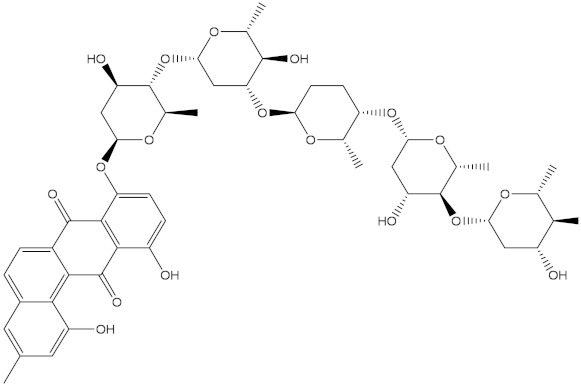	−11.3	−11.2	NPA001007 (Landomycin U)	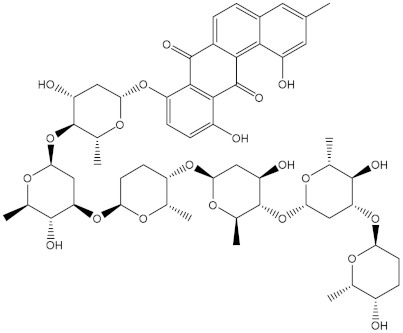	−10.8	−10.9
NPA003114 (Fradimycin B)	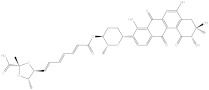	−11.1	−11.1	NPA032617 (Taichunamide B)	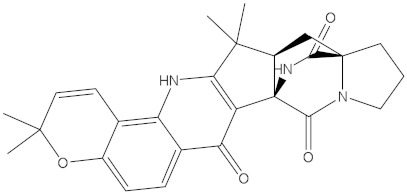	−10.8	−10.8
NPA022085 (Versiquinazoline C)	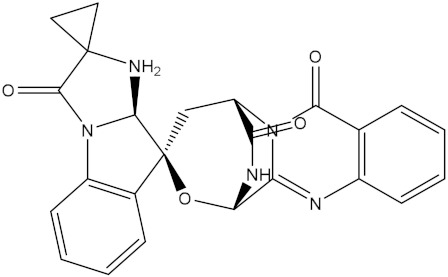	−11.1	−11.1	NPA032380 (Mangrovamide A)	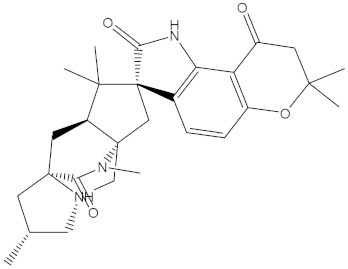	−10.8	−10.8
NPA016707 (Galtamycin B)	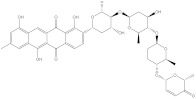	−11.4	−11.1	NPA032307 (Waikikiamide B)	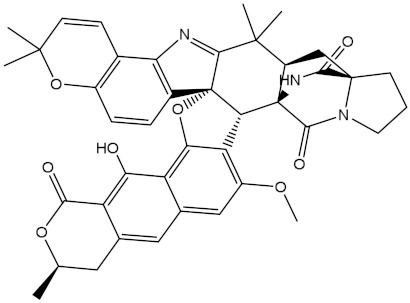	−10.8	−10.8
NPA031305 (Chloroxaloterpin A)	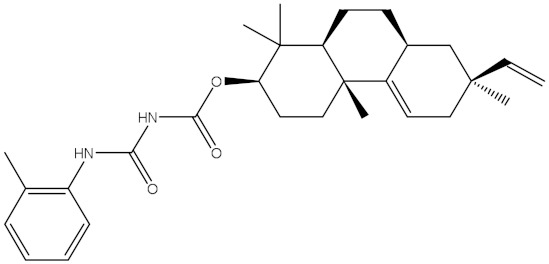	−11.0	−11.0	NPA010784 (Stephacidin A)	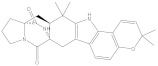	−10.7	−10.8
NPA024299 (Asperversiamide B)	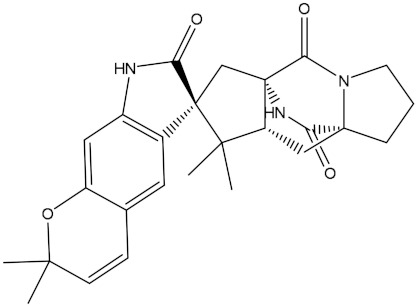	−11.0	−11.0	NPA008251 (Landomycin Q)	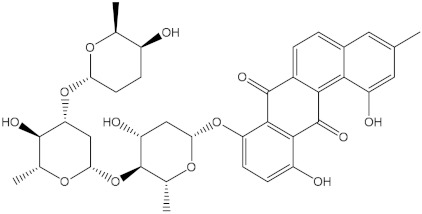	−10.9	−10.8
NPA018626 (Sterhirsutin H)	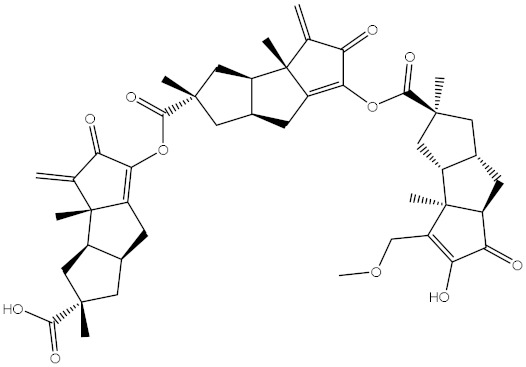	−11.0	−11.0	NPA002417 (Landomycin V)	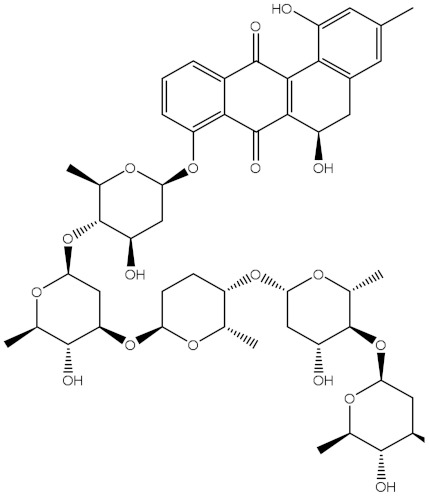	−10.8	−10.
NPA013855 (CJ-17665)	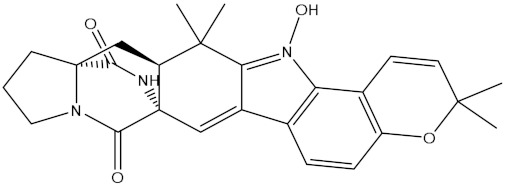	−10.9	−11.0	NPA025114 (Tomophagusin A)	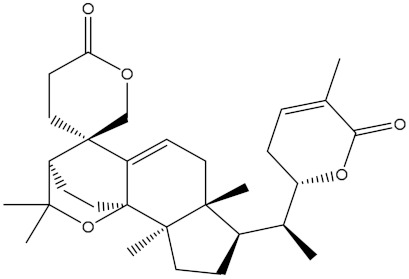	−10.7	−10.7
NPA032618 (Taichunamide C)	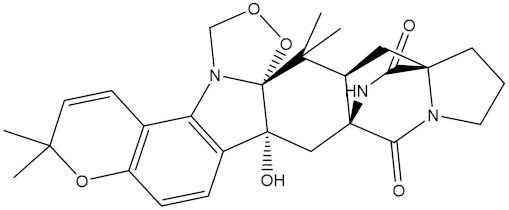	−10.9	−10.9	NPA014914 (5-chlorosclerotiamide)	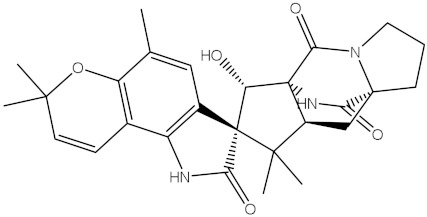	−10.7	−10.7
NPA020206 ((+)-6-epi-stephacidin A)	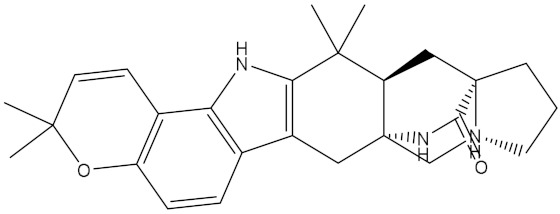	−10.9	−10.9	NPA012265 (N05WA963A)	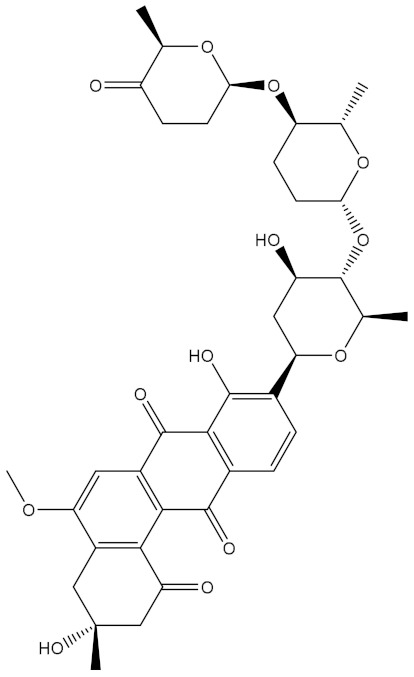	−10.7	−10.7
NPA010050 (Shearinine H)	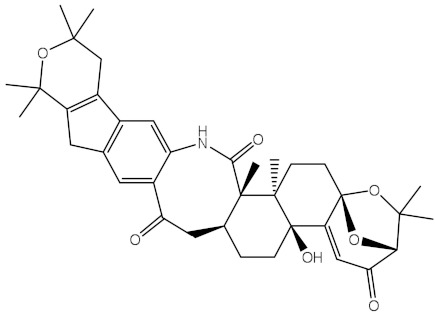	−10.9	−10.8	NPA012190 (Paraherquamide K)	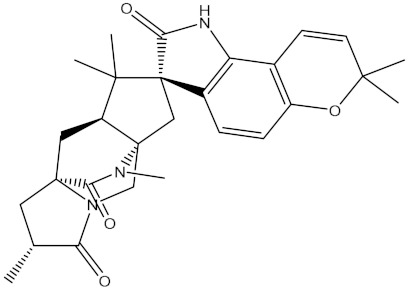	−10.7	−10.7
NPA011832 (Sterhirsutin I)	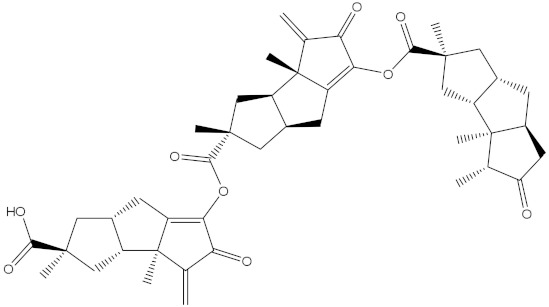	−10.7	−10.7	NPA004417 (29-N-demethylparaherquamide K)	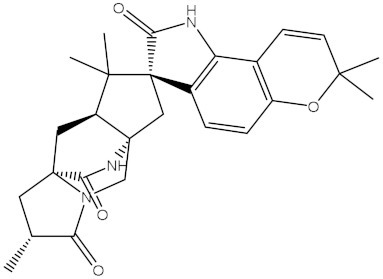	−10.7	−10.7
NPA010595 (Neocitreamicin II)	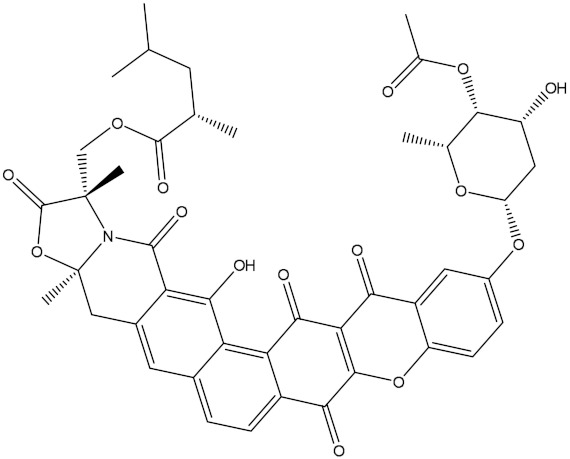	−10.7	−10.7	NPA004402 (Notoamide I)	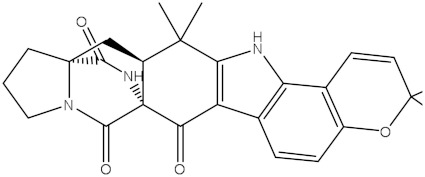	−10.7	−10.7
NPA007920 ((+)naseseazine A)	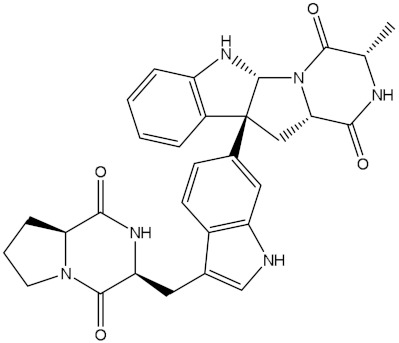	−10.7	−10.7	NPA003296 (Ganodermalactone G)	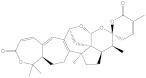	−10.7	−10.7
NPA006989 (PI-087)	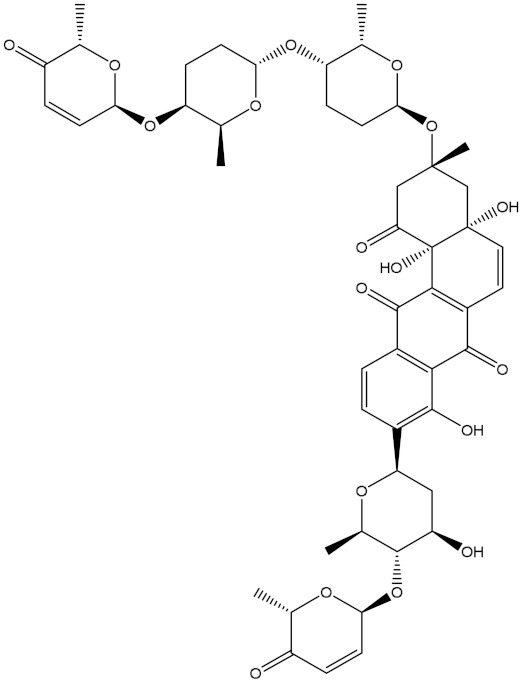	−10.7	−10.7	NPA001446 (Austamide)	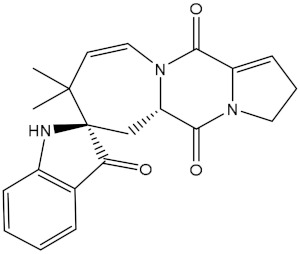	−10.7	−10.7
NPA006530 (N05WA963D)	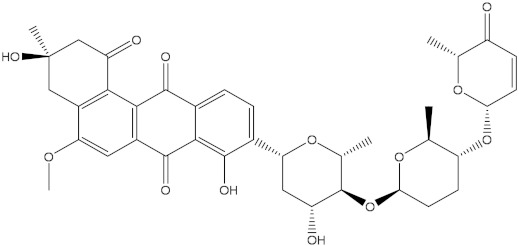	−10.7	−10.7				

^a^ Data ranked based on the expensive docking scores.

**Table 2:** The calculated MM-GBSA binding energies of venetoclax and saquayamycin F with the Bcl-2 protein over 50 and 200 ns MD simulations.

NPAtlas Code	MM-GBSA Binding Energy (kcal/mol)	
	50 ns	200 ns
Venetoclax	–46.0	−50.6
Saquayamycin F (NPA002200)	–48.1	–53.9

**Table 3:** Predicted physiochemical properties of saquayamycin F (NPA002200) and venetoclax as Bcl-2 inhibitors.

Compound Name/Code	miLog *P*	TPSA	nON	nOHNH	Nrotb	MVol	MW	%ABS
Venetoclax	8.43	174.72	14	3	13	758.49	868.46	48.7
Saquayamycin F (NPA002200)	0.65	230.91	16	4	7	715.32	822.86	29.3

**Table 4:** The predicted ADMET characteristics for venetoclax and the saquayamycin F (NPA002200).

Compound Name/Code	Absorption (A)		Distribution (D)	Metabolism (M)	Excretion (E)	Toxicity (T)
	Caco2 Permeability	Human Intestinal Absorption (HIA)	Log BB	CYP3A4 Inhibitor/Substrate	Total Clearance	AMES Toxicity
Venetoclax	0.847	100	–1.9	Yes	–0.096	No
Saquayamycin F (NPA002200)	0.271	80.9	0.27	Yes	–0.196	No

## Data Availability

The data presented in this study are available in the Supplementary Materials.

## Supplementary Materials

The following supporting information can be downloaded at: https://www.mdpi.com/article/10.3390/molecules28020783/s1, Figure S1. 2D representations of the anticipated binding modes for the top 42 NPAtlas compounds inside the active site of the Bcl-2 protein; Figure S2. Three-dimensional molecular interactions of (a) saquayamycin F (NPA002200) and (b) venetoclax with the Bcl-2 protein according to the final snapshot over 50 ns MD simulations. Table S1: Calculated fast and expensive docking scores (in kcal/mol) for venetoclax and the top 56 NPAtlas compounds against the Bcl-2 protein; Table S2: Estimated fast, expensive docking scores, and MM-GBSA binding energies (in kcal/mol) over 50 ns MD simulations for venetoclax and the top 42 NPAtlas compounds within the Bcl-2 protein.
