# Supplementary material for: In Silico Mining of Natural Products Atlas (NPAtlas) Database for Identifying Effective Bcl-2 Inhibitors: Molecular Docking, Molecular Dynamics, and Pharmacokinetics Characteristics
Source: Molecules. 2023 Jan 12;28(2):783. doi: 10.3390/molecules28020783 (PMC9864825; doi:10.3390/molecules28020783)
Supplement: Supplementary file 1 [file molecules-28-00783-s001.zip › molecules-2134983-supplementary.pdf]

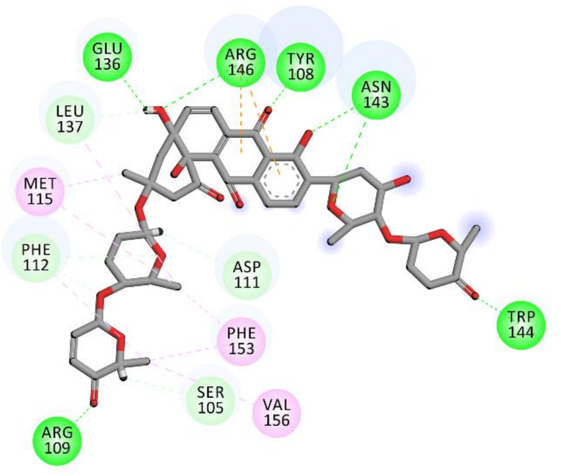

NPA002200

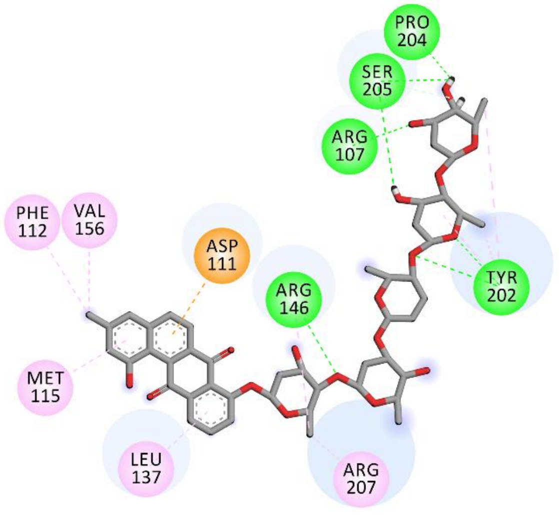

NPA032668

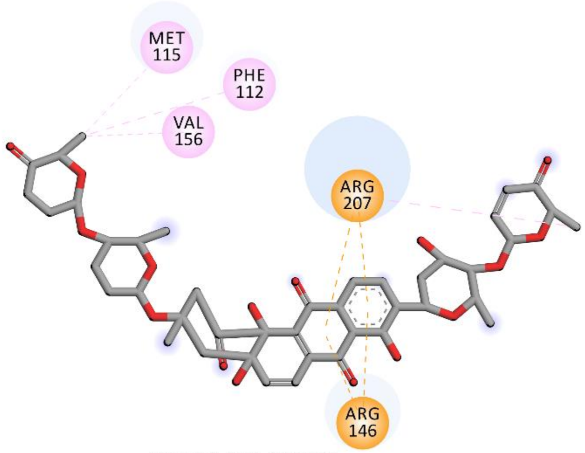

NPA004880

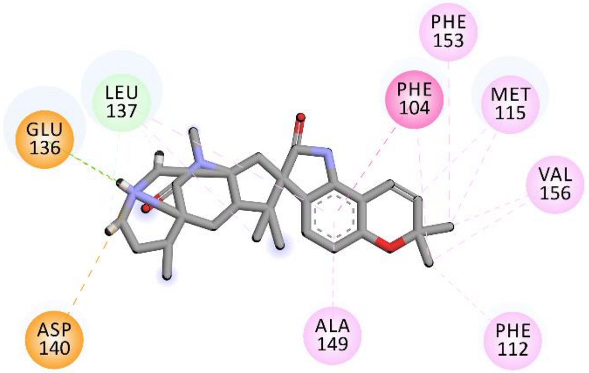

NPA021302

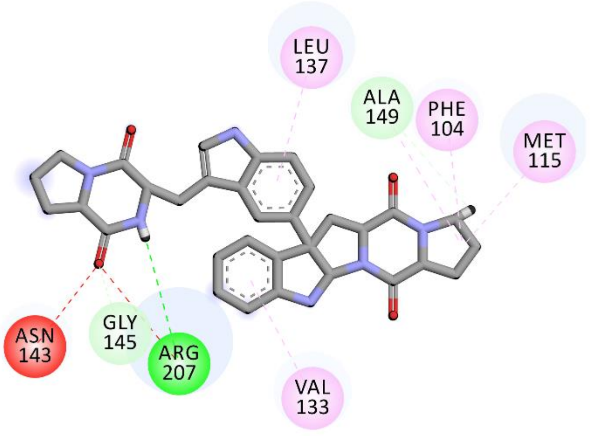

NPA008122

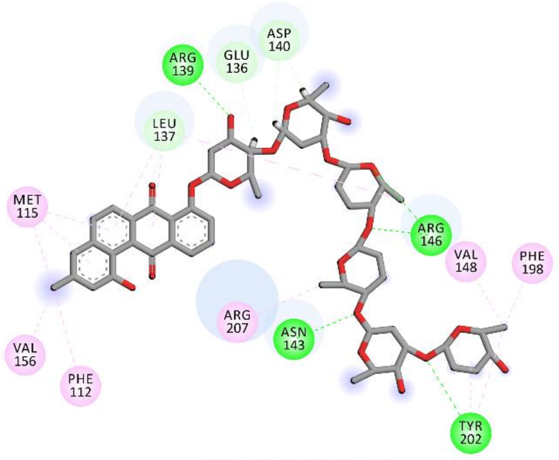

NPA018272

Figure S1. 2D representations of the anticipated binding modes for the top 42 NPAtlas compounds inside the active site of the Bcl-2 protein.

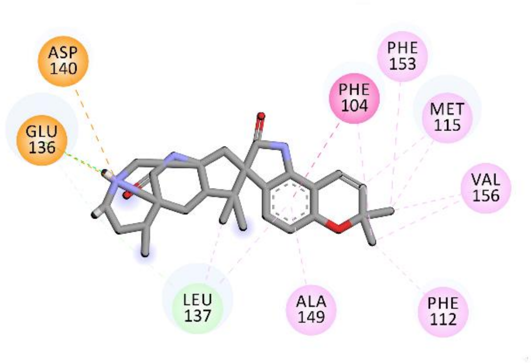

NPA012375

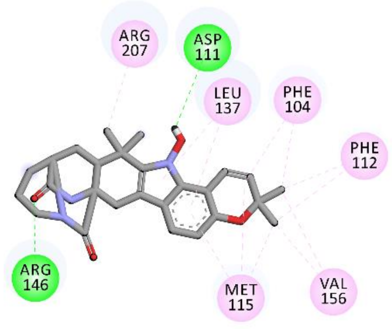

NPA008326

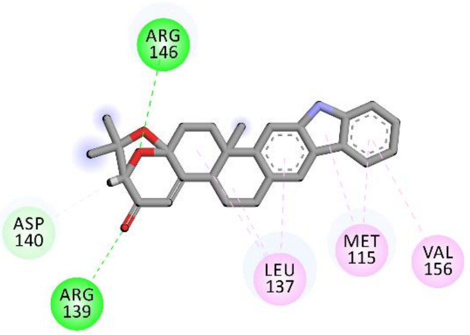

NPA025253

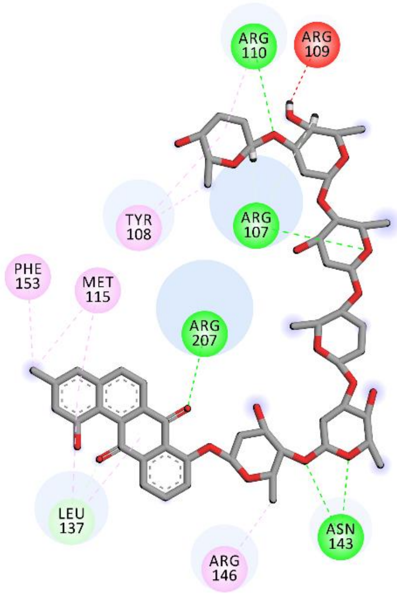

NPA019494

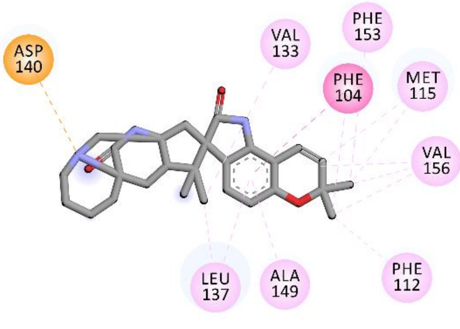

NPA005301

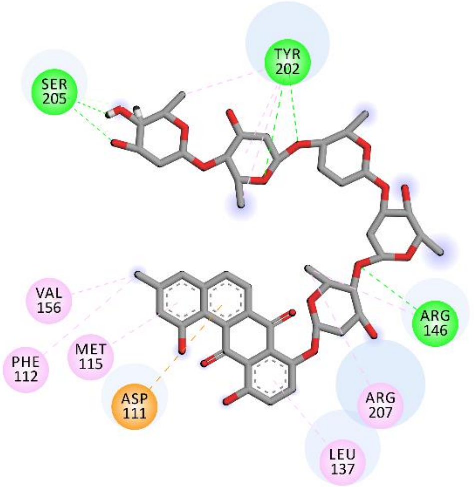

NPA005183

Figure S1. *Continued.*

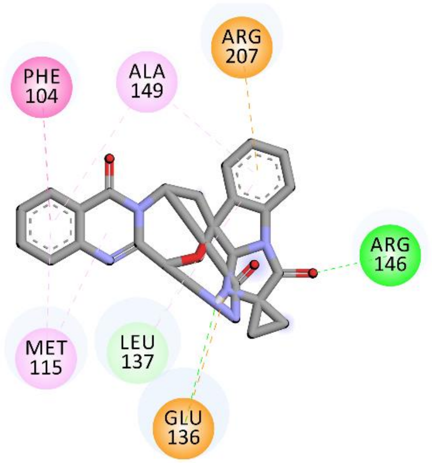

NPA022085

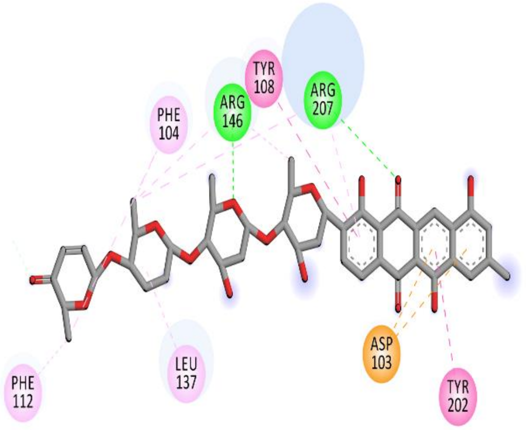

NPA016707

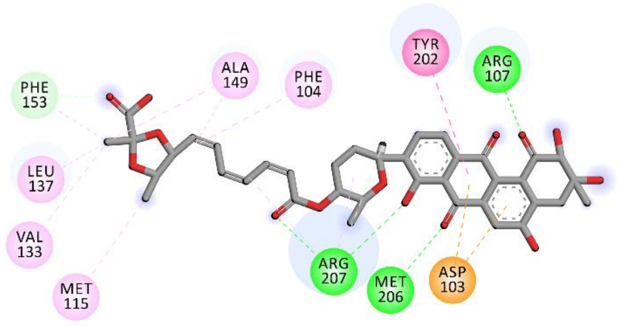

NPA003114

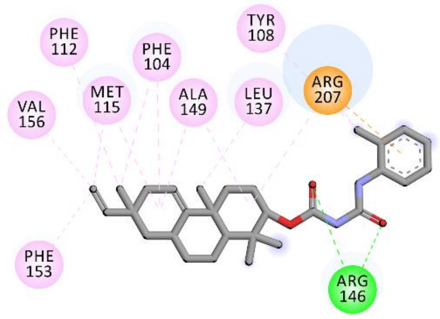

NPA031305

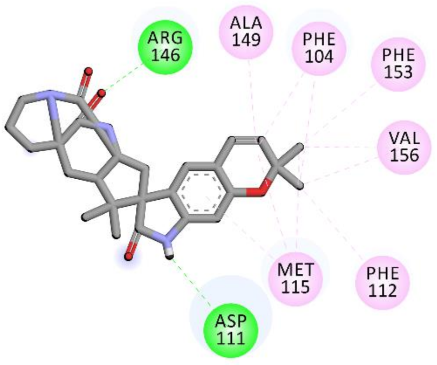

NPA024299

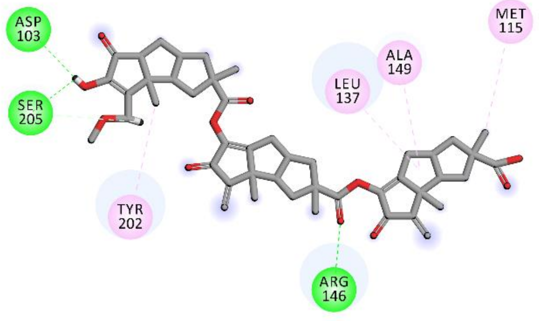

NPA018626

Figure S1. *Continued.*

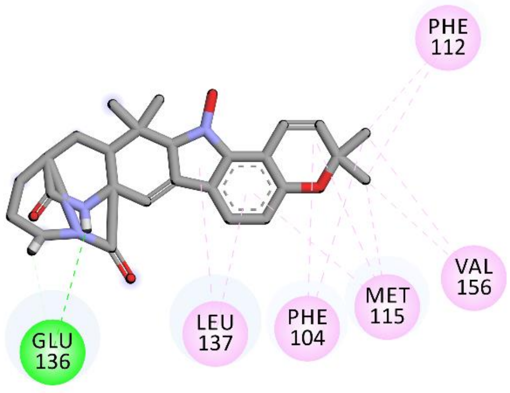

NPA013855

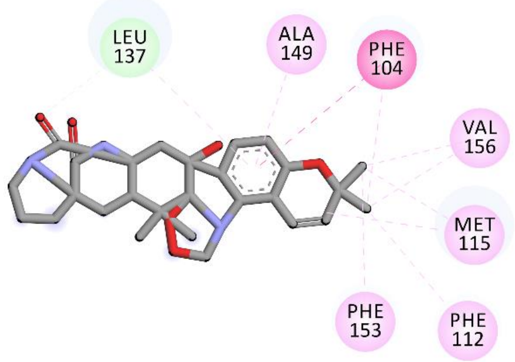

NPA032618

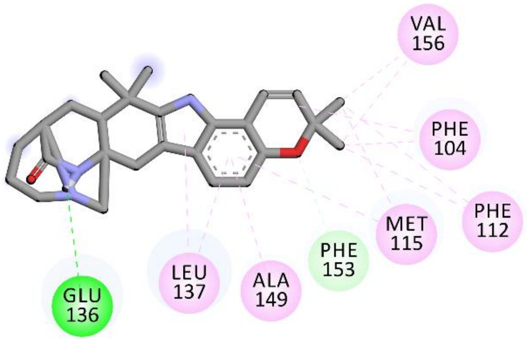

NPA020206

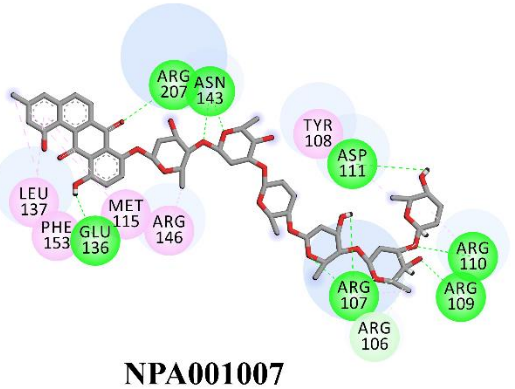

NPA001007

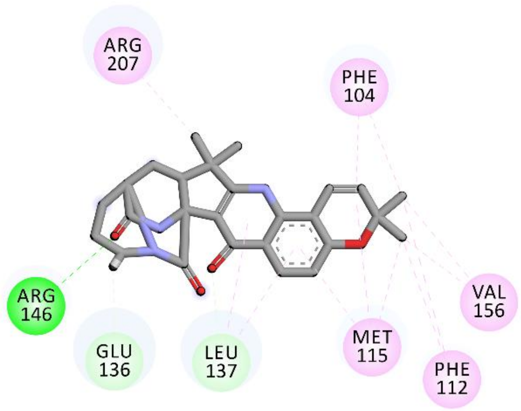

NPA032617

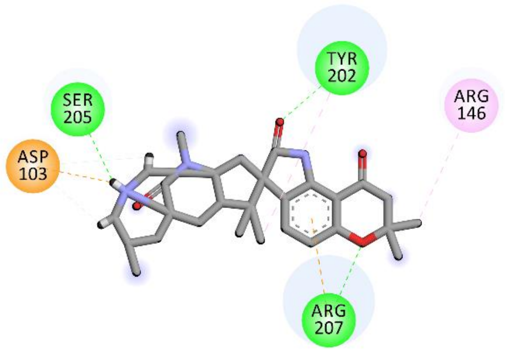

NPA032380

Figure S1. *Continued.*

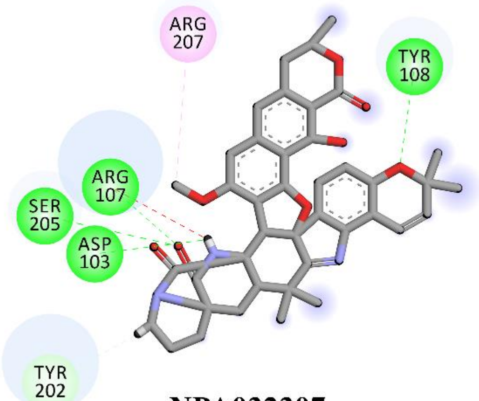

NPA032307

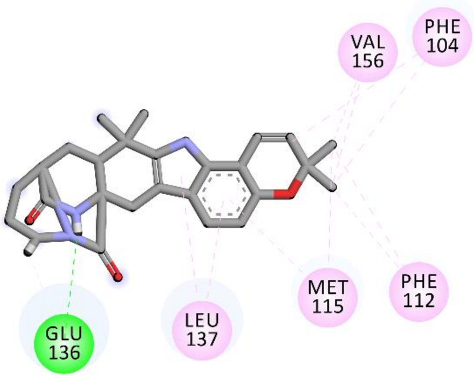

NPA010784

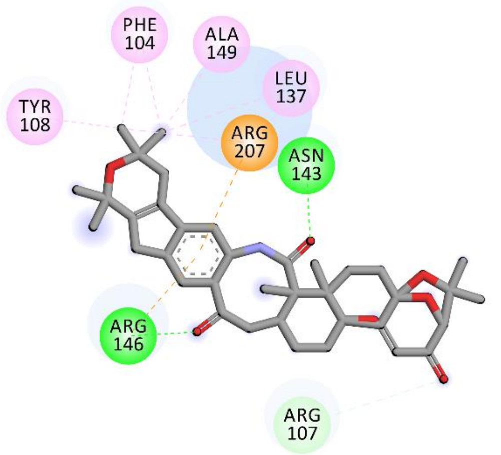

NPA010050

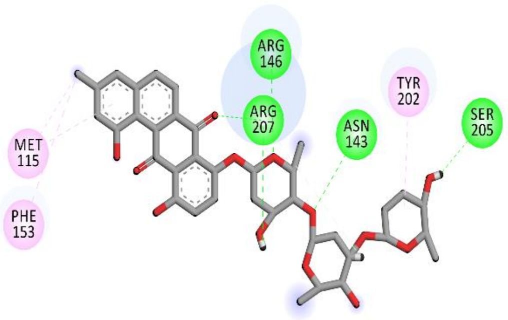

NPA008251

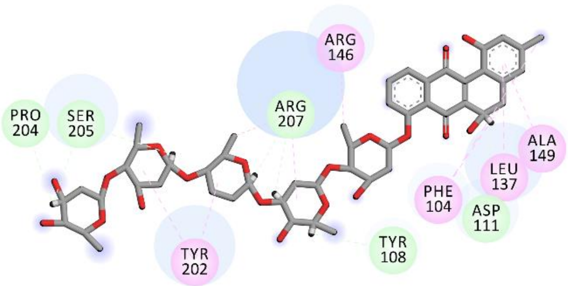

NPA002417

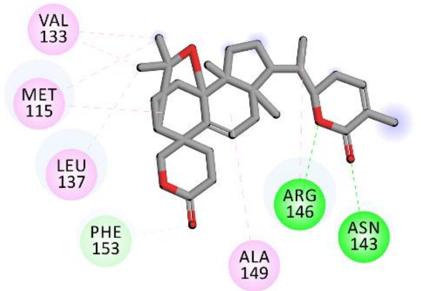

NPA025114

Figure S1. *Continued.*

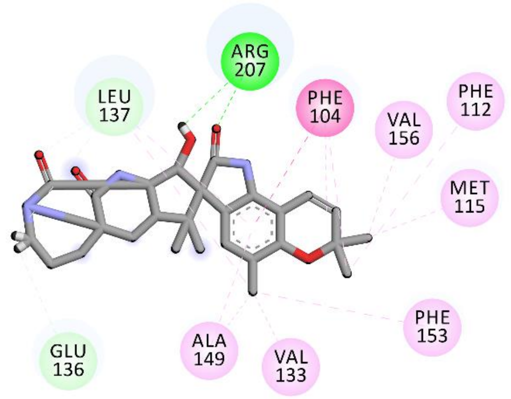

NPA014914

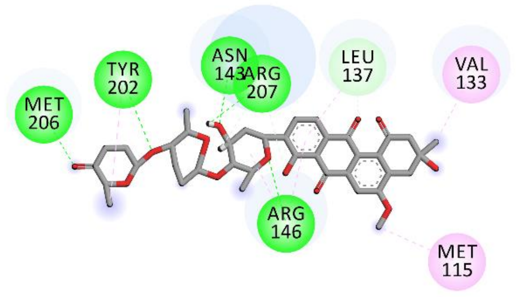

NPA012265

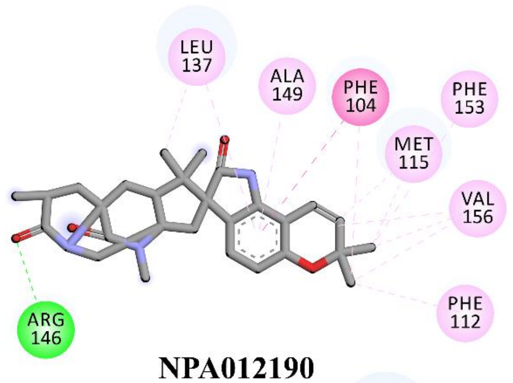

NPA012190

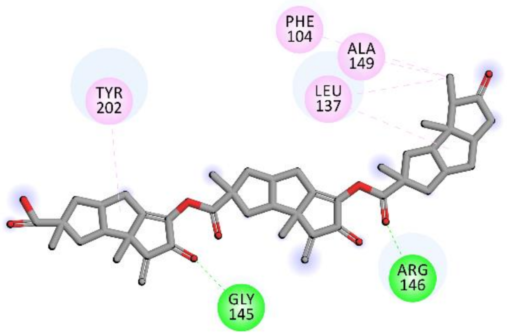

NPA011832

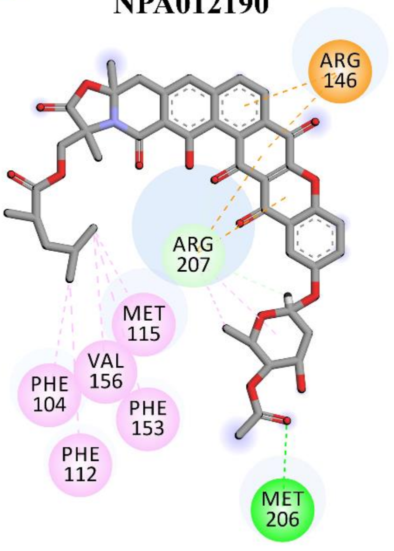

NPA010595

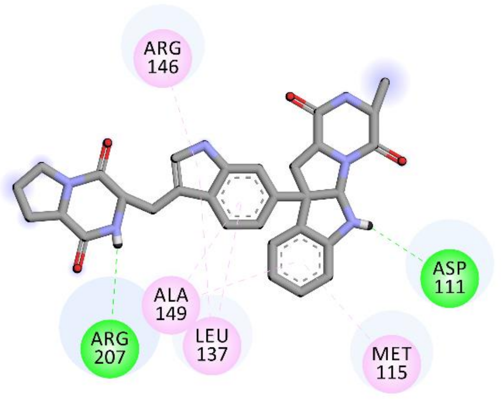

NPA007920

Figure S1. *Continued.*

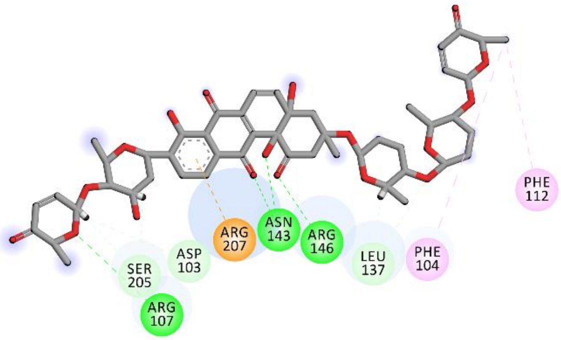

NPA006989

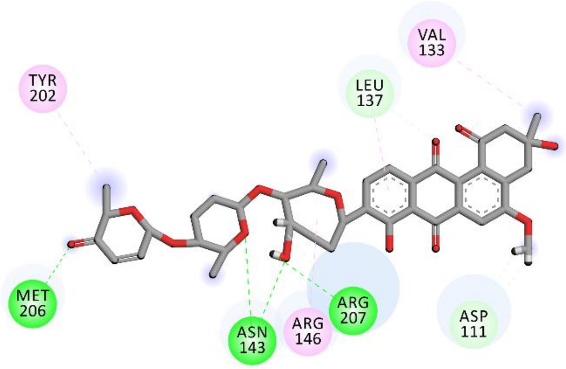

NPA006530

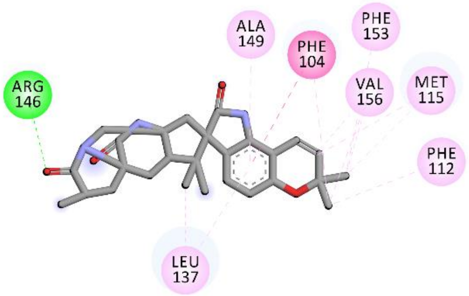

NPA004417

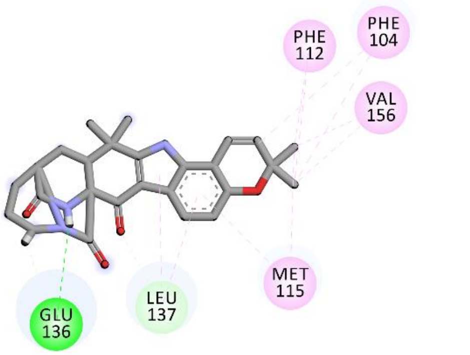

NPA004402

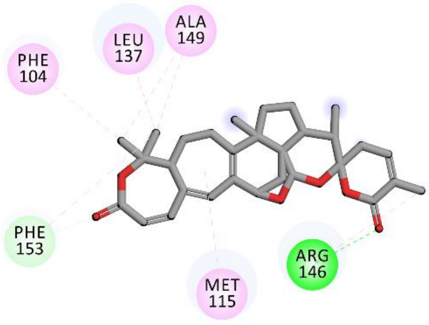

NPA003296

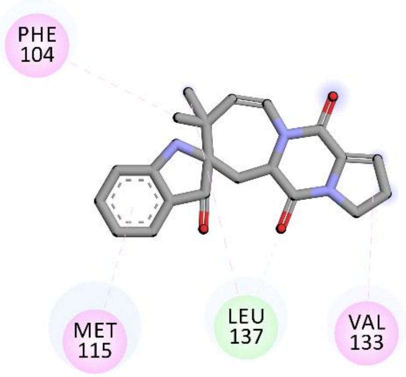

NPA001446

Interactions

- Conventional Hydrogen Bond
- Carbon Hydrogen Bond
- Pi-Doner Hydrogen Bond
- Unfavorable Acceptor-Acceptor
- Unfavorable Doner-Doner
- Alkyl
- Pi-Alkyl
- Pi-Pi Stacked
- Pi-Pi T-shaped
- Attractive Charge
- Pi-Anion
- Pi-Cation

Figure S1. *Continued*.

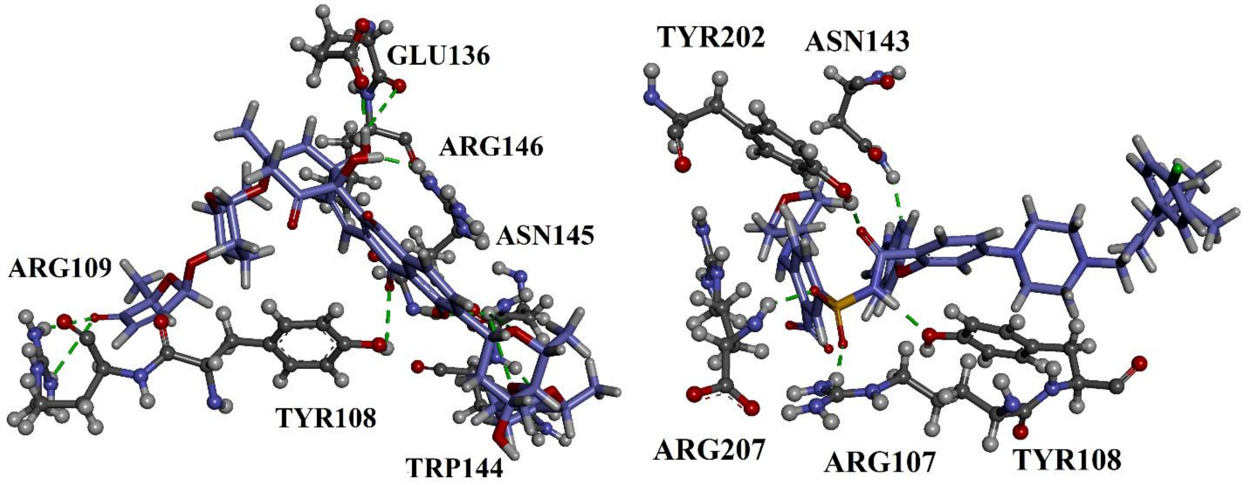

(a) Saquayamycin F (NPA002200) **(b) Venetoclax**

Figure S2. Three-dimensional molecular interactions of (a) saquayamycin F (NPA002200) and (b) venetoclax with the Bcl-2 protein according to the final snapshot over 50 ns MD simulations.

Table S1. Calculated fast and expensive docking scores (in kcal/mol) for venetoclax and the top 56 NPAtlas compounds against the Bcl-2 protein.[a]

No.	NPAtlas Code	Docking Score (kcal/mol)		No.	NPAtlas Code	Docking Score (kcal/mol)	
		Fast	Expensive			Fast	Expensive
	**Ventoclax**	–10.6	–10.6	29	NPA032617	–10.8	–10.8
1	NPA002200	–12.0	–12.0	30	NPA006989	–10.7	–10.7
2	NPA032668	–11.7	–11.8	31	NPA025114	–10.7	–10.7
3	NPA004880	–11.7	–11.7	32	NPA007920	–10.7	–10.7
4	NPA021302	–11.6	–11.6	33	NPA012265	–10.7	–10.7
5	NPA008122	–11.5	–11.5	34	NPA003296	–10.7	–10.7
6	NPA012375	–11.4	–11.4	35	NPA006530	–10.7	–10.7
7	NPA018272	–11.3	–11.4	36	NPA004417	–10.7	–10.7
8	NPA025253	–11.3	–11.3	37	NPA014914	–10.7	–10.7
9	NPA008326	–11.3	–11.3	38	NPA004402	–10.7	–10.7
10	NPA005301	–11.1	–11.2	39	NPA001446	–10.7	–10.7
11	NPA019494	–11.2	–11.2	40	NPA010595	–10.7	–10.7
12	NPA005183	–11.3	–11.2	41	NPA011832	–10.7	–10.7
13	NPA022085	–11.1	–11.1	42	NPA012190	–10.7	–10.7
14	NPA003114	–11.1	–11.1	43	NPA003513	–10.7	–10.6
15	NPA016707	–11.4	–11.1	44	NPA005062	–10.7	–10.6
16	NPA013855	–10.9	–11.0	45	NPA012585	–10.7	–10.6
17	NPA018626	–11.0	–11.0	46	NPA014280	–10.7	–10.6
18	NPA024299	–11.0	–11.0	47	NPA018173	–10.7	–10.6
19	NPA031305	–11.0	–11.0	48	NPA020806	–10.7	–10.6
20	NPA020206	–10.9	–10.9	49	NPA021382	–10.7	–10.6
21	NPA001007	–10.8	–10.9	50	NPA021659	–10.7	–10.6
22	NPA032618	–10.9	–10.9	51	NPA022641	–10.7	–10.6
23	NPA008251	–10.9	–10.8	52	NPA022806	–10.7	–10.6
24	NPA010050	–10.9	–10.8	53	NPA025468	–10.7	–10.6
25	NPA002417	–10.8	–10.8	54	NPA031950	–10.7	–10.6
26	NPA032307	–10.8	–10.8	55	NPA000445	–10.7	–10.5
27	NPA010784	–10.7	–10.8	56	NPA010907	–10.7	–10.5
28	NPA032380	–10.8	–10.8				

[a]Data ranked based on the expensive docking scores.

Table S2. Estimated fast, expensive docking scores, and MM-GBSA binding energies (in kcal/mol) over 50 ns MD simulations for venetoclax and the top 42 NPAtlas compounds within the Bcl-2 protein.[a]

No.	NPASS Code	Docking Score (kcal/mol)		MM-GBSA Binding Energy (kcal/mol)
		Fast	Expensive	
	**Ventoclax**	–10.6	–10.6	–46.0
1	NPA002200	–12.0	–12.0	–48.1
2	NPA005301	–11.1	–11.2	–41.7
3	NPA006989	–10.7	–10.7	–41.3
4	NPA025114	–10.7	–10.7	–40.2
5	NPA016707	–11.4	–11.1	–39.2
6	NPA019494	–11.2	–11.2	–39.1
7	NPA007920	–10.7	–10.7	–38.6
8	NPA008251	–10.9	–10.8	–37.1
9	NPA032618	–10.9	–10.9	–37.0
10	NPA012375	–11.4	–11.4	–36.7
11	NPA021302	–11.6	–11.6	–35.4
12	NPA013855	–10.9	–11.0	–35.0
13	NPA031305	–11.0	–11.0	–34.8
14	NPA012265	–10.7	–10.7	–34.6
15	NPA008326	–11.3	–11.3	–34.3
16	NPA018626	–11.0	–11.0	–33.5
17	NPA010595	–10.7	–10.7	–33.2
18	NPA005183	–11.3	–11.2	–32.9
19	NPA020206	–10.9	–10.9	–32.7
20	NPA001007	–10.8	–10.9	–31.9
21	NPA032668	–11.7	–11.8	–31.8
22	NPA011832	–10.7	–10.7	–30.8
23	NPA024299	–11.0	–11.0	–30.7
24	NPA010784	–10.7	–10.8	–30.1
25	NPA003296	–10.7	–10.7	–29.6
26	NPA025253	–11.3	–11.3	–29.4
27	NPA018272	–11.3	–11.4	–29.2
28	NPA006530	–10.7	–10.7	–28.9
29	NPA004417	–10.7	–10.7	–28.4
30	NPA014914	–10.7	–10.7	–28.2
31	NPA004402	–10.7	–10.7	–27.9
32	NPA022085	–11.1	–11.1	–27.6
33	NPA008122	–11.5	–11.5	–27.3
34	NPA012190	–10.7	–10.7	–27.1
35	NPA003114	–11.1	–11.1	–26.3
36	NPA032617	–10.8	–10.8	–25.5
37	NPA004880	–11.7	–11.7	–25.3
38	NPA001446	–10.7	–10.7	–25.3
39	NPA010050	–10.9	–10.8	–24.7
40	NPA002417	–10.8	–10.8	–23.4
41	NPA032380	–10.8	–10.8	–13.6
42	NPA032307	–10.8	–10.8	–1.8

[a]Data ranked based on the MM-GBSA binding energy over the 50 ns MD simulations.
